# Quantitative analysis of changes in movement behaviour within and outside habitat in a specialist butterfly

**DOI:** 10.1186/1471-2148-7-4

**Published:** 2007-01-22

**Authors:** Nicolas Schtickzelle, Augustin Joiris, Hans Van Dyck, Michel Baguette

**Affiliations:** 1 Biodiversity Research Centre, University of Louvain (UCL), 4 Place Croix du Sud, B-1348 Louvain-la-Neuve, Belgium; 2 Muséum National d'Histoire Naturelle, Département Ecologie et Gestion de la Biodiversité, UMR CNRS-MNHN 7179, Rue du petit château, 4, F-91800 Brunoy, France

## Abstract

**Background:**

Dispersal between habitat patches is a key process in the functioning of (meta)populations. As distance between suitable habitats increases, the ongoing process of habitat fragmentation is expected to generate strong selection pressures on movement behaviour. This leads to an increase or decrease of dispersal according to its cost relative to landscape structure. To limit the cost of dispersal in an increasingly hostile matrix, we predict that organisms would adopt special dispersal behaviour between habitats, which are different from movements associated with resource searching in suitable habitats.

**Results:**

Here we quantified the movement behaviour of the bog fritillary butterfly (*Proclossiana eunomia*) by (1) assessing perceptual range, the distance to which the habitat can be perceived, and (2) tracking and parameterizing movement behaviour within and outside habitat (parameters were move length and turning angles distributions). Results are three-fold. (1) Perceptual range was < 30 m. (2) Movements were significantly straighter in the matrix than within the habitat. (3) Correlated random walk adequately described movement behaviour for 70% of the observed movement paths within habitat and in the matrix.

**Conclusion:**

The perceptual range being lower than the distance between habitat patches in the study area, *P. eunomia *likely perceives these habitat networks as fragmented, and must locate suitable habitats while dispersing across the landscape matrix. Such a constraint means that dispersal entails costs, and that selection pressure should favour behaviours that limit these costs. Indeed, our finding that dispersal movements in the matrix are straighter than resource searching movements within habitat supports the prediction of simulation studies that adopting straight movements for dispersal reduces its costs in fragmented landscapes. Our results support the mounting evidence that dispersal in fragmented landscapes evolved towards the use of specific movement behaviour, different from explorative searching movements within habitat.

## Background

Dispersal, the movement of organisms between spatial units of habitat, is an essential life-history trait shaping the characteristic texture of populations, communities and ecosystems in space and time. Despite the numerous books and papers on the subject ([1-6] among several others), dispersal remains one of the least understood factors in evolutionary biology, especially the behavioural mechanisms that underlie this complex phenomenon [7].

Dispersal has been defined as a three-stage process: (1) emigration (crossing habitat boundaries), (2) travelling through a more or less hostile landscape matrix, and (3) immigration (settlement in a new habitat) [8]. When the degree of habitat fragmentation increases, the distances to be covered through the landscape increase as well, which may result in dispersal-related deferred costs (i.e. lower fitness of immigrants due to loss of time and energy during travel in the matrix) or even mortality (e.g. [9,10]). Hence, we may predict that behavioural responses have evolved to limit costs and risks of dispersal. Indeed, empirical studies show that organisms are more reluctant to cross the border of the habitat (i.e. where they find resources to complete their life cycle) in fragmented landscapes (e.g. [11,12]). This was particularly clear in our study species, the bog fritillary butterfly (*Proclossiana eunomia*), a habitat specialist species restricted to unfertilized alluvial wet meadows and peat bogs [13,14]. This behavioural response affects the first stage of dispersal, i.e. emigration.

Changes in behaviour during travel in the matrix, the second stage of dispersal, can be another adaptive answer to reduce the cost of dispersal. We recently suggested that dispersal can be realized in two ways: as a by-product of routine movements associated with resource exploitation (like foraging or mate-searching) with high levels of returning, or as special, fast and directed movements designed for net displacement [7]. We also predict that the latter, directed flight behaviour should occur more frequently in fragmented landscapes as it allows maximizing the probability to end up in another unit of habitat by limiting travelling time across the landscape matrix (e.g. [13,15,16]). A simulation model [17] predicts that evolution towards straighter movement paths is to be expected both when (1) dispersing individuals travel through a more hostile landscape and (2) inter-habitat distances increase. Several empirical studies show variation in dispersal behaviour relative to landscape structure (e.g. [18-27]). Most of those studies investigated dispersal in the landscape without reference to movement behaviour within the habitat (but see [28]) and there are only few quantitative analyses.

**Figure 1:**
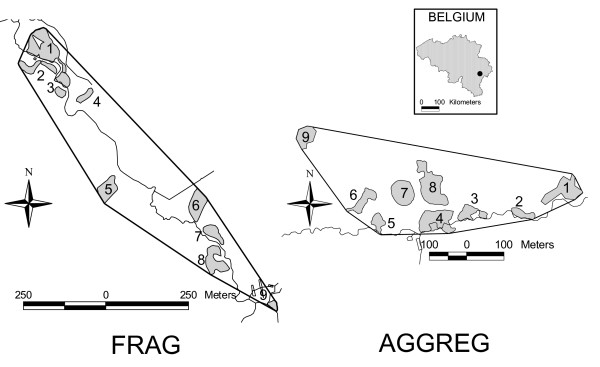
**Map of the study sites in southern Belgium**. Favourable habitat (wet meadows or peat bogs with *Polygonum bistorta*) is delineated in grey; the rest of the landscape is unsuitable and called the matrix. Patches 1 and 9 did not exist in the AGGREG network at the time of previous studies of *P. eunomia*, including the within-habitat movement study [14].

In this paper, we test for differences between movement behaviour within habitat and outside habitat (i.e. in the matrix) in two habitat networks of southern Belgium (Figure 1), one largely fragmented (denoted FRAG), the other more aggregated (denoted AGGREG). Assuming that specialist butterflies perceive their environment as fragmented (i.e. individuals are not able to perceive other suitable habitat patches when they leave their current patch), we hypothesize that dispersal movements in the matrix are relatively straight whereas search movements when foraging within habitat are rather tortuous. Habitat fragmentation in the study area occurred since > 50 years or butterfly generations [29]. Such a time frame allows evolutionary changes to habitat fragmentation in flying insects [30].

Experimental evidence indicates that butterflies respond to visual and/or olfactory cues to locate landscape elements and to detect suitable habitat [31]. We first quantified the distance component of the perceptual range (i.e. the distance at which the habitat is detected: [32,33]) to test the assumption that the study landscapes were perceived as fragmented by *P. eunomia *butterflies. Next, we recorded movement paths of butterflies in the matrix and compared movement parameters to those previously recorded within habitat [14], to test our prediction that movements are straighter in the matrix. Finally, we assessed whether movement behaviour in the matrix may be adequately described by a correlated random walk. This would allow the use of simulation models of movement to predict dispersal at the metapopulation level.

## Results

### Perceptual range

**Figure 2:**
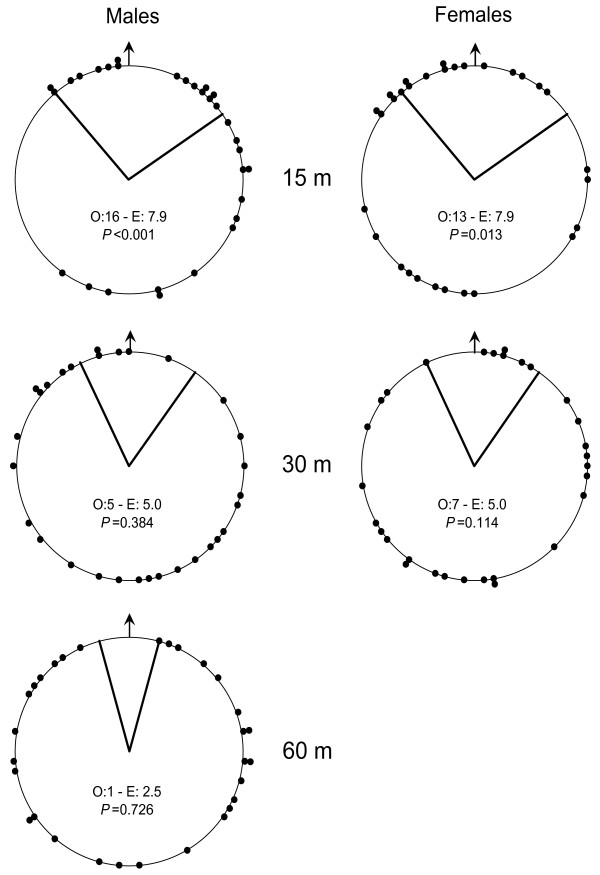
**Orientation of butterflies towards the habitat**. Starting direction of butterflies released in the AGGREG landscape matrix (i.e. outside habitat) at 15, 30 and 60 m of the habitat. Each dot represents one release (30 releases per distance and sex); lines around the 0° (arrow) delimit the angles directed towards the habitat patch. O: observed numbers of individuals orientating towards the patch; E: expected numbers under hypothesis of random starting direction; *P*: p-value for the one-tailed binomial test of orientation towards the habitat patch *vs*. random [54 p. 533]. No female was released at 60 m to limit the impact of lost females on reproduction rate in this protected and threatened species.

We measured perceptual range by assessing the maximum distance at which butterflies could orientate towards the habitat when released in the matrix (i.e. outside suitable habitat). *P. eunomia *adults (both males and females) showed a highly significant response to return into the habitat patch when released at 15 m of the patch. This response completely vanished at wider distance: at 30 and 60 m of the patch they flew at random in all directions (Figure 2). Perceptual range of *P. eunomia *was therefore estimated to be below 30 m.

### Movement behaviour

**Figure 3:**
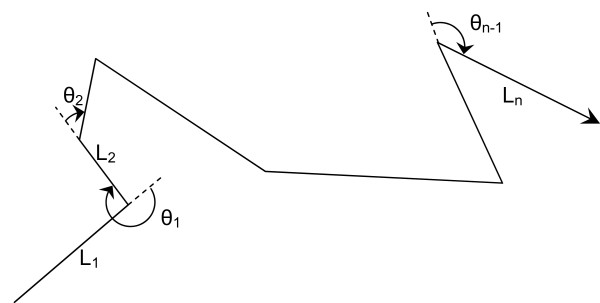
**Schematic representation of a movement path**. Movement paths are approximated by a sequence of straight line moves and turning angles. L_i_: length of the i^th ^move; θ_i_: turning angle between moves i and i + 1.

Adults were tracked individually to record movement paths. Every stop or turn was marked by a numbered flag, and coordinates mapped by triangulation. Movements were approximated as sequences of straight lines moves and turning angles (Figure 3). In the habitat (data published in [14]), we recorded 17 female paths (223 positions) in FRAG and 36 female paths (524 positions) in AGGREG. In the matrix (new data collected for the present study), we recorded 42 male paths (625 positions) in FRAG, 15 male paths (117 positions) and 15 female paths (155 positions) in AGGREG. Differences in numbers mainly reflect different weather conditions affecting sampling opportunities via their effect on butterfly abundance (highly variable from year to year: [34]) and responsible for tracking interruptions.

#### Movement parameters in habitat and matrix

**Figure 4:**
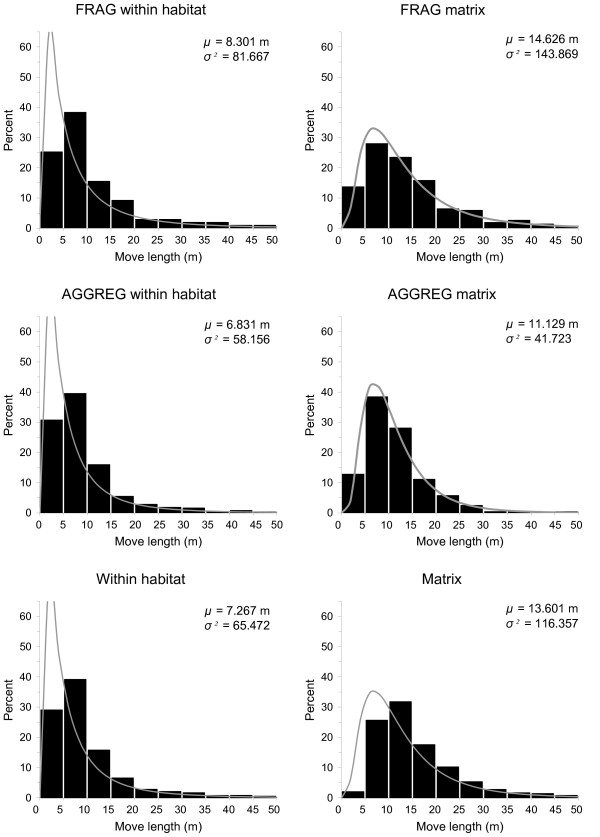
**Distributions of move length**. Distribution of move length for movements within habitat and in the matrix in the two landscapes, showing that movements were longer and straighter in the matrix. Black: observed (a few observations longer than 50 m in the matrix have been omitted). Grey curve: fitted lognormal distribution with mean *μ *and variance *σ*^*2*^.

**Figure 5:**
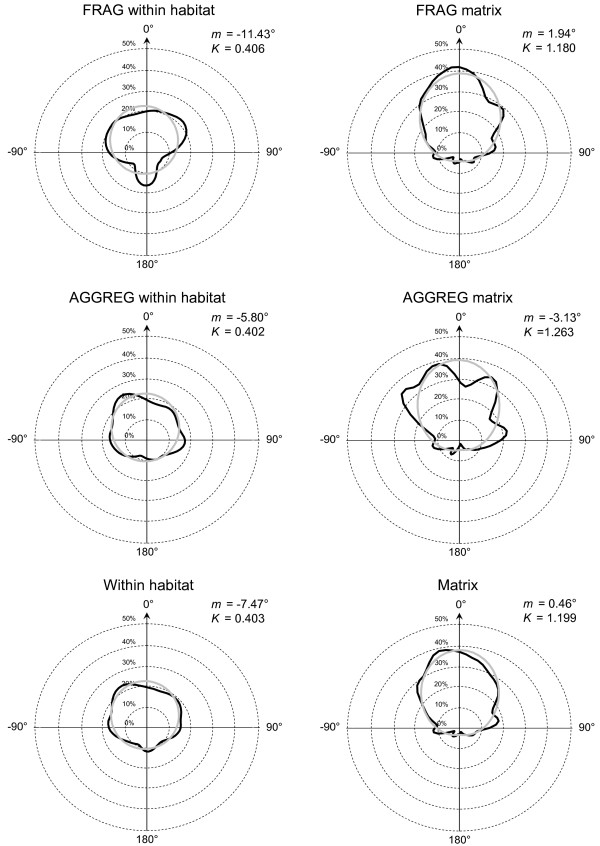
**Distributions of turning angle**. Distribution of move length for movements within habitat and in the matrix in the two landscapes, showing that movements were straighter (smaller turning angles) in the matrix. Black: observed (kernel density estimate using h = 0.5*h0, i.e. a kind of moving average: [53] p. 24 *ff*). Grey curve: fitted von Mises (i.e. circular normal) distribution with mean *m *and concentration *K*.

Whatever the habitat type and the landscape, movement parameters fit the same distribution: a lognormal distribution for move length (Figure 4, Table 1), with frequent short and rare long moves between successive stops/turns, and a symmetrical around zero von Mises distribution for turning angle (Figure 5, Table 2): butterflies did not show a preference for left or right turns and made more often small than large turns.

**Table 1:** Statistical tests of differences in move length (log-transformed) for habitat vs matrix and FRAG vs AGGREG.

Test 1: Goodness-of-fit for lognormal distribution: *D *(*P*)			
	FRAG	AGGREG	Pooled
Habitat	0.050 (> 0.150)	0.018 (> 0.150)	0.023 (> 0.150)
Matrix	0.024 (> 0.150)	0.056 (0.067)	0.029 (0.087)
			
Test 2: Equality of means *μ*: 2-way ANOVA			
Factor	*F*_1;1515_	*P*	

Habitat type	295.33	< 0.0001	
Landscape	10.04	0.002	
Interaction	0.17	0.677	
			
Test 3: Equality of variances σ^*2*^: Bartlett: χ_1_^2 ^(*P*)			

	FRAG	AGGREG	Pooled
Habitat	3.061 (0.080)		129.900 (< 0.0001)
Matrix	16.928 (< 0.0001)		

Note: Test numbers refer to Table 4, where more details on tests and references are available. Legend of table content is given in corresponding test title. While it is possible to obtain full test of the two factors and their interaction at once for *μ *using 2-factor ANOVA, there is no such test for σ^*2*^. Therefore, in this case, we decided to compare groups in two steps: first the landscape comparing FRAG *vs *AGREGG for each habitat type separately, then the environment type (our major interest) comparing habitat patch *vs *matrix on landscape-pooled data.

**Table 2:** Statistical tests of differences in turning angle for habitat vs matrix and FRAG vs AGGREG.

Test I: Uniform *vs *unimodal distribution with mean *m *= 0: *R *(*P*)			
	FRAG	AGGREG	Pooled
Habitat	0.195 (0.0002)	0.196 (< 0.0001)	0.196 (< 0.0001)
Matrix	0.509 (< 0.0001)	0.533 (< 0.0001)	0.516 (< 0.0001)
			
Test II: Symmetrical distribution around 0: *W*^+ ^(*P*)			

	FRAG	AGGREG	Pooled
Habitat	-0.207 (0.836)	1.442 (0.149)	1.009 (0.313)
Matrix	0.295 (0.768)	0.900 (0.368)	0.797 (0.425)
			
Test III: Goodness-of-fit for von Mises distribution: *U*^2 ^(*P*)			

	FRAG	AGGREG	Pooled
Habitat	0.045 (0.200)	0.056 (0.100)	0.039 (0.300)
Matrix	0.032 (> 0.500)	0.094 (0.035)	0.036 (> 0.500)
			
Test IV: von Mises mean *m *= 0: *E*_*n *_(*P*)			

	FRAG	AGGREG	Pooled
Habitat	-0.775 (0.438)	-0.605 (0.545)	-0.929 (0.353)
Matrix	0.609 (0.542)	-0.652 (0.514)	0.174 (0.862)
			
Test V: Equality of von Mises concentrations *K*: *f*_*r *_(*P*)			

	FRAG	AGGREG	Pooled
Habitat	0.127 (0.722)		10.821 (< 0.0001)
Matrix	0.132 (0.725)		

Note: Test numbers refer to Table 5, where more details on tests and references are available. Legend of table content is given in corresponding test title.

**Figure 6:**
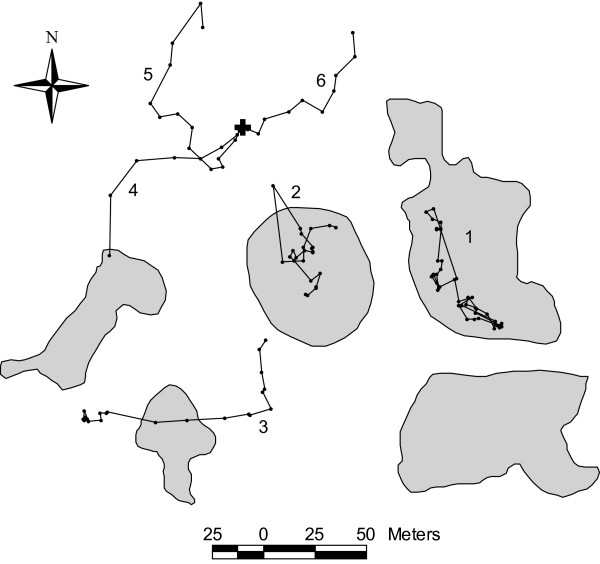
**Maps of selected paths in AGGREG illustrating the main findings**. The major conclusion is that butterflies made tortuous paths (small moves interrupted by frequent and large turns) when flying within habitat (path 1), but flew straighter (long moves, small turns) in the matrix (paths 4, 5 and 6). Path 2 shows the tendency to return into the patch after having crossed the habitat patch boundary [14]. Path 3 resulted from a spontaneous flight (without experimental manipulation) and illustrates that experimental artefacts due to manipulation were very unlikely the cause for straighter paths in the matrix (see Methods for details). Path 4 illustrates the return to a suitable habitat patch, with the last turn at a distance of c. 25 m from the patch, well around the estimate of perceptual range. Grey: habitat patches; +: release site in the matrix.

Moves were on average twice as long in the matrix as in the habitat (Figure 4, Table 1). They were similar for FRAG and AGGREG within habitat, but slightly longer and more variable in FRAG matrix than in AGGREG matrix. More importantly, there was an obvious difference of path linearity between matrix and habitat: (1) the concentration parameter *K *was much higher in the matrix while nearly absolutely identical in the two networks (Figure 5, Table 2); (2) the fractal dimension was lower in the matrix while here again nearly identical in FRAG and AGGREG (Table 3); and (3) for a given number of moves, the net displacement was larger in the matrix (Figure 6).

**Table 3:** Statistical tests of differences in fractal dimension for habitat vs matrix and FRAG vs AGGREG.

	FRAG	AGGREG	Pooled
Habitat	1.2641 (1.2318–1.2965)	1.2882 (1.2343–1.3422)	1.2805 (1.2431–1.3180)
Matrix	1.1525 (1.1318–1.1732)	1.1576 (1.1346–1.1806)	1.1546 (1.1396–1.1696)

Note: Significance is determined by no overlap of 95% confidence interval (given into brackets)

#### Goodness-of-fit to correlated random walk

There were no differences in the frequency of departures from Correlated Random Walk (CRW) between FRAG and AGGREG, nor between habitat and matrix (Fisher's exact test all *P *> 0.05). Overall, the majority of the paths (around 70% in both habitat types) were well described by CRW, only 3% (4/125, 1 in habitat and 3 in matrix) are underpredicted by CRW (too straight movements), 26% (32/125, 16 in habitat and 16 in matrix) overpredicted by CRW (returns towards a previously visited area, mainly due to the existence of some boundaries to dispersal, like tree lines). CRW is an adequate description of movement paths both within and outside habitat; differences in linearity are achieved by distributions of move length and turning angle differing between habitat and matrix.

## Discussion

Our results support four main conclusions. (1) The perceptual range of *P. eunomia *is relatively low. Hence, it confirms the assumption that butterflies perceived both habitat networks as fragmented. (2) The behavioural nature of movements differs significantly between habitat and matrix: butterflies were found to fly straighter (longer distances, smaller turns) in the matrix (Figure 6). (3) Movements differed between FRAG and AGGREG landscapes in the matrix, but not within habitat. (4) Movement behaviour can be adequately modelled by a correlated random walk (CRW), both within habitat and in the matrix.

### Perceptual range is low

Our experiments indicated a relatively low perceptual range in *P. eunomia*, between 15 and 30 m only. As a consequence, both the FRAG and AGGREG landscapes arise as fragmented habitat networks for these butterflies, i.e. individuals are not able to perceive other suitable habitat patches when they leave their current patch. This perceptual range is in agreement with the few other studies existing on butterflies: 10–22 m in *Icaricia icarioides fenderi *[12], a few meters in *Pieris napae *[35], 8 m in *Phoebis sennae *and *Eurema nicippe *[36]. Of course, our experiment does not allow discriminating between the two alternatives that butterflies were unable or unmotivated to orientate towards the habitat patch. Indeed, we observed some butterflies flying towards a habitat patch from a distance longer than 30 m, but this may be done by chance only. The finding that orientation towards habitat patch is done from a distance far lower than the distance individuals are able to disperse successfully -*P. eunomia *is able to disperse between relatively distant patches (> several kilometres: [13,37,38])- has also been observed in *Euphydryas editha bayensis *[39], and *Pararge aegeria *[40]. If habitat detection is visually cued, this is in line with the 20–30 m distance of large object detection predicted from optical properties of butterfly eyes [41]. Alternatively, olfactory clues may be used to orientate towards habitat. Both senses can also be involved [42,43]. Navigation clues used during dispersal require further mechanistic research, as well as parallel studies on perceptual range of species with different habitat requirements.

### Dispersal flights in the matrix are straighter movements than search flights within habitat

Both networks being perceived as fragmented by butterflies, selection pressure due to costs of dispersal are likely to occur. We showed previously that mortality during dispersal was higher in FRAG than in AGREG [13,14]. Therefore, we predicted the use of specific dispersal behaviours limiting these costs. Accordingly, within-habitat movements differed from those in the matrix. Several lines of evidence showed that paths were relatively straight in the matrix while largely tortuous within habitat. The same conclusion was drawn for both FRAG and AGGREG landscapes, and these two landscapes present similar path linearity for a given environmental condition (habitat or matrix). This observation is in line with the view of [7] that special dispersal movements may differ behaviourally from routine, explorative movements associated with resource (food, mate, etc.) searching. Our results are also in line with predictions of simulation models [17], where relatively straight CRWs have been shown to be among the best search strategies to find habitat patches over long distances in fragmented landscapes, especially when perceptual range is small compared to the average between-patch distance. It is worth noting that in landscapes where habitat patches are highly aggregated, the reverse is expected: more tortuous movements are expected to increase dispersal success [17,44].

### Moves in matrix are longer in the more fragmented landscape

Differences in movement behaviour between FRAG and AGGREG landscapes were clearly small compared to those between matrix and habitat. We previously showed that movements did not differ between landscapes within habitat, except for behaviour at habitat patch boundaries [14]. The present results complete the picture for movements in the matrix: linearity was similar in the two landscapes but moves were longer in FRAG. This latter result might be related to fragmentation level (higher in FRAG) and the nature of the matrix (more hostile in FRAG). We have previously shown that mortality during dispersal was higher than mortality within habitats [13,14], which should favour individuals with a higher dispersal success, notably through longer and straighter moves as discussed above. On the other hand, there is a growing body of evidence that variation in landscape heterogeneity affects movement behaviour in animals, including butterflies (e.g. [23,24,26,45-48]). Nevertheless, we acknowledge that, given the present experimental design with one replicate per fragmentation level (often inevitable in practice), this difference between FRAG and AGGREG matrices (and only this one) could also be due to another particular difference between landscapes.

### Movement behaviour of *P. eunomia *can be adequately modelled as a correlated random walk

Movement behaviour could be adequately modelled in 70% of the cases by a correlated random walk (CRW) with lognormal move length and von Mises (circular normal) turning angles; these distributions are environment-specific (matrix vs habitat) (Figure 4, Figure 5), which allows modelling both tortuous movements in the habitat and straight movements in the matrix under the same CRW framework. The most frequent departures from CRW were caused by disruptions in the path due to the presence of some barriers to dispersal (e.g. habitat patch boundaries in habitat, tree lines in matrix), which reflect individuals towards their point of origin [14]. These departures do not alter the adequacy of CRW to model movement in barrier-free zones.

## Conclusion

Dispersing individuals suffer costs from mortality, or fitness depletion due to a loss of time and energy on the way between habitats. Those costs are expected to shape adaptive answers on dispersal behaviours in highly fragmented landscapes where distance between suitable habitats increases. We showed previously that dispersal propensity, i.e. the probability that an individual decided to cross habitat boundaries to engage the dispersal process, decreased according to the fragmentation level of the landscape. This was proved to be associated with an increase in the mortality risk associated to dispersal. Here we show that dispersal in two fragmented landscapes was achieved by special directed movements significantly straighter and longer than those movements that are associated with resource searching within habitat. We showed previously that even if the overall dispersal mortality was higher in fragmented than in continuous landscapes, *for a given patch connectivity *mortality was lower in more fragmented landscapes [13]. This is likely associated to the use of straighter flight in the matrix, a good strategy to improve dispersal success.

## Methods

### Species

The bog fritillary (*Proclossiana eunomia *ESPER) is a habitat specialist butterfly living in unfertilized alluvial wet meadows and peat bogs. In Western Europe, these are the only places where the unique food plant of larvae and adults (the bistort *Polygonum bistorta *L.) grows in reasonable densities [49,50]. Modern land-use has transformed considerable amounts of former habitat into improved or overgrown pastures, or into spruce plantations. Consequently, the amount of remaining suitable habitat decreased and the degree of fragmentation increased [29]. It is a vulnerable species in Western Europe and is legally protected in the study area (Wallonia, S-Belgium).

### Study systems

*P. eunomia *occurs as metapopulations in the Plateau des Tailles upland in southern Belgium [38]. We previously selected two habitat networks showing different levels of fragmentation to study the movement behaviour within suitable habitat [14] (Figure 1). The fragmented network (FRAG) is the Prés de la Lienne nature reserve, where *P. eunomia *has a metapopulation distributed among nine habitat patches (in total *c*. 2.3 ha of suitable habitat) in a highly fragmented landscape. The edge-to-edge mean inter-patch distance to closest neighbour is 54 m with a range of 5–222 m [34,37]. The more aggregated network (AGGREG) is situated in the Pisserotte nature reserve. It sustains a metapopulation distributed among nine habitat patches (*c*. 2.5 ha of suitable habitat; mean edge-to-edge inter-patch distance to closest neighbour 23 m, range 13–37 m) in a natural and relatively undisturbed peat bog. The main difference between the two sites lies in the fragmentation level (higher in FRAG) and the nature of the matrix: cultivated land in FRAG (mainly fertilized pastures), natural vegetation in AGGREG (areas of the peat bog that lack the host plant). Both networks are isolated from other metapopulations in the landscape [13].

### Measuring perceptual range

We measured perceptual range (sensu Euclidian detection distance: [33]) by assessing the maximum distance at which butterflies could orientate towards the habitat; this experiment was conducted during the 2004 flight season (June). Adults were netted one at a time, kept in the net and released a few minutes later in the matrix at 15, 30 and 60 m of the habitat patch. Butterflies were carefully placed on a natural substrate. Usually, they spent some time basking before taking off spontaneously. Several precautions were taken to ensure the reliability of measurements and prevent artefacts that could originate from three sources. (1) Releases were done only when weather conditions were favourable, i.e. when many butterflies were observed flying; weather conditions (air temperature and wind speed) during releases were recorded and proved not to influence orientation nor differ between release distances (not shown). (2) Releases were randomized between sexes and distances to prevent confusion of these factors with weather conditions. (3) Butterflies were marked to control for pseudoreplication arising from the repeated use of the same individuals.

The overall starting flight direction (averaged during the first 5 m) of each individual was recorded using a compass. Orientation ability at a given distance from the habitat was inferred when the proportion of butterflies starting towards the habitat patch was significantly higher than expected if they would fly randomly. The expected number of butterflies starting towards the patch under the hypothesis of no orientation was computed as the number of releases (30) times the proportion of the circle oriented towards the patch, and compared to the observed number using Chi square tests.

### Mapping and quantifying movement behaviour

Each adult was followed until it was lost from sight or rested for 15 min. Every stop or turn was marked by a numbered flag. During tracking, disturbance was avoided by following the butterflies from a distance. Adults are only rarely observed leaving a patch of habitat. Therefore to record sufficient numbers of flight paths in the matrix, adults were netted in a habitat patch nearby and immediately released in the matrix using the same standard method as for measuring perceptual range; the same requirements of favourable weather conditions were applied.

Coordinates of all the stops/turns in each path were subsequently determined by triangulation using measured distances to three reference points of known coordinates. To allow a quantitative statistical analysis of movement parameters, flight paths were represented as sequences of straight-line moves (between two consecutive turns/stops) and associated turning angles (Figure 3). This is widely used to represent animal movements in random walk models ([51] and references therein). Analysis was done with two major aims: (1) estimation and comparison of movement parameters between the habitat and the matrix, and (2) goodness-of-fit (GOF) test to correlated random walk (CRW). Unless otherwise specified, all data management and analyses were done using SAS software with home-written programs [52].

#### Estimation and comparison of movement parameters in habitat and matrix

Three path variables were analyzed: move length, turning angle (direction and amplitude), and path linearity. Move length and turning angles were pooled for all paths of a group and analyzed by statistical methods appropriate for linear [53,54] and circular data ([55] and references therein; see also [54] chapters 26–27), respectively (See Table 4 and Table 5 for a full description of the tests and references). Firstly, we assessed the GOF to a specific distribution: lognormal for move length, von Mises for turning angle (i.e. the circular equivalent of the Normal distribution for linear quantities: [[55] p. 48–56]). Secondly, we tested for equality of distribution moments between groups: mean *μ *and variance σ^*2 *^of log-transformed move-length, mean *m *and concentration *K *of turning angle (*K *is analogous to the variance of a linear distribution but varies inversely: with higher *K*, angles are more concentrated around the mean). The third variable to be analyzed, the linearity (or inversely the "tortuousity") of the path, was evaluated by the fractal dimension *d *(a straight line has *d *= 1, and *d *increases as the linearity of the path decreases: [56]) using the VFractal software [57].

**Table 4:** Statistical tests of differences in move length between males and females.

	Null hypothesis tested	Test name	Test statistic	*P*	Reference*
Test 1a	Goodness-of-fit for lognormal distribution: females	Kolmogorov-Smirnov	*D *= 0.071	0.083	[53] (pp. 708–715)
Test 1b	Goodness-of-fit for lognormal distribution: males		*D *= 0.054	> 0.150	
Test 2	Equality of means *μ*_*i *_between sexes	1-way ANOVA (on log-transformed move length)	*F*_*1;240 *_= 0.58	0.449	[53] (pp. 272–320)
Test 3	Equality of variances σ^*2*^_*i *_between sexes	Bartlett (on log-transformed move length)	χ_1_^2 ^= 2.11	0.146	[53] (pp. 396–401)

* Original and complementary references can be found there.

**Table 5:** Statistical tests of differences in turning angle between males and females.

	Null hypothesis tested	Test name	Test statistic	*P*	Reference*
Test Ia	Uniform *vs *unimodal distribution with mean *m *= 0: females	Modified Rayleigh test for uniformity against a unimodal alternative with mean = 0	*R *= 0.602	< 0.0001	[55] (p. 69)
Test Ib	Uniform *vs *unimodal distribution with mean *m *= 0: males		*R *= 0.433	< 0.0001	
Test IIa	Symmetrical distribution around 0: females	Wilcoxon signed-rank test	*W*^+ ^= 0.747	0.455	[55] (pp. 80–81)
Test IIb	Symmetrical distribution around 0: males		*W*^+ ^= 0.154	0.877	
Test IIIa	Goodness-of-fit for von Mises distribution: females	Watson's *U*^2^	*U*^2 ^= 0.115	0.020	[69]†
Test IIIb	Goodness-of-fit for von Mises distribution: males		*U*^2 ^= 0.070	0.085	
Test IVa	von Mises mean *m *= 0: females	Unnamed specific test	*E*_*n *_= -0.182	0.856	[55] (pp. 93–94)
Test IVb	von Mises mean *m *= 0: males		*E*_*n *_= -0.782	0.434	
Test V	Equality of von Mises concentrations *K*_*i *_between sexes	Fisher *f*_*r*_; *p *value estimated by permutation test	*f*_*r *_= 3.861	0.052	[55] (pp. 131–132)

* Original and complementary references can be found there.

† [55] (p. 84) presents a mistake.

#### Goodness-of-fit (GOF) to correlated random walk

To test for the GOF to correlated random walk (CRW), we used another measure of linearity: the net squared displacement (i.e. square of the distance between position after *n *moves and starting point: *R*^*2*^_*n*_; [51,58]). The more linear the path, the steeper is the increase of *R*^*2*^_*n *_with *n*. Using a bootstrap procedure [59] based on 1000 simulations per path, we assessed whether each path could be explained by specific movement rules defining CRW: move length follows a lognormal distribution and turning angle follows a von Mises distribution, both with parameters estimated in homogeneous groups of paths defined by the analysis of these variables. If *R*^*2*^_*n *_is significantly too small, it indicates the tendency to return (i.e. barrier effect on movement), whereas a *R*^*2*^_*n *_that is significantly too high indicates a movement straighter than average CRW.

#### Assessment of possible artefacts due to experimental design

As far as experimental design is concerned there is a clear trade-off for experimental evolutionary ecological field studies between rarity/threat status of study species and the experimental freedom or flexibility. Several researchers opt for working with common species that easier allow a balanced experimental design in the field. However, we share the view of [60] that studies on rare species are nevertheless important to ecological and evolutionary theory. In particular, *P. eunomia *is a narrow habitat specialist, making it more reliable to define habitat and non-habitat. This is, however, often more difficult -but frequently neglected- in more common species that find ample but often dispersed resources across the landscape matrix [61]. Hence, a perfect experimental design but a less reliable distinction of habitat vs non-habitat categories demonstrates that even then some weakness may remain in such a study. The issue of habitat vs matrix is extremely clear-cut in the case of our study species *P. eunomia *[62].

*P. eunomia *is a threatened and protected species of clear conservation concern that is mainly confined to nature reserves in our study area [49,50]. This status limits the application of some potentially harmful experiments. As in all our previous studies on *P. eunomia*, we decided to minimise harmful experiments when we were intimately convinced that the knowledge we had on the species would allow maintaining the high quality standard we require with less harmful experiments, even if those were suboptimal in terms of experimental design. We therefore address here potential artefacts in our experimental design, and discuss why these were inevitable in the present field study though unlikely to confound our conclusions. Potential artefacts relate to (1) the flight motivation of butterflies outside suitable habitat and the possible influence of netting and translocating, (2) the potential of sex-related biases in the observations, (3) the recording of turns and not only stops in the matrix, and (4) the comparison of data recorded in different years.

Individuals released outside habitat were netted and induced to fly whereas they were not necessarily motivated to do so. Hence, one could argue that the results do not reflect natural dispersal behaviour. Butterflies leave only rarely habitat patches during observation sessions at habitat borders. This was in any case far too rare to allow proper statistical analysis. Although netting and releasing may in principle have caused changes in individual behaviour ([63], but see e.g. [64]), we argue, based on our long-term experience with butterfly behaviour after capture (15 years for *P. eunomia *in the Prés de la Lienne study site: [13] and references therein), that this was at the utmost limited. For instance during capture-mark-recapture studies, some individuals were netted several times within a timeframe of a few minutes as it was not possible to detect any behavioural difference between recently caught and other individuals. In some other butterfly species, escape behaviour has occasionally been observed after being marked, while in again some others there is often a substantial recovery period in the vegetation after being marked [65,66]. Moreover, we did also observe a few *P. eunomia *individuals flying naturally in the matrix, of which two were recorded when tracking butterflies within AGGREG habitat [14], as they started in the habitat. The flight behaviour of these "free flying" butterflies, which were neither netted nor translocated, was in all aspects similar to the behaviour of released ones (see path 3 on Figure 6); but too few such paths were available to allow statistical testing to confirm the similarity.

In the habitat, only females were tracked because of the biological importance of female movements spreading eggs: in such a polygynous species the reproduction rate in the population mainly depends on laying rate by females, male abundance being likely a non-limiting factor [14]. In the matrix, both sexes were tracked in AGGREG, while only males were tracked in FRAG to avoid a significant impact on reproduction rate in this limited population following the potential death of females in case they were definitely lost in the matrix after release. There was no difference between sexes in terms of movement parameters, for both move length (Table 4) and turning angle (Table 5). Furthermore, the existing differences between habitat and matrix were even more pronounced when the comparison was achieved by keeping only data for the same sex (females in AGGREG; not shown). Hence, our comparison is conservative in this respect and the risk of spurious effects due to the sex is considered extremely limited.

Using stops instead of sampling at fixed time intervals is biologically more meaningful. Each stop corresponds to a decision made by the individual. It prevents problems of over- or undersampling due to the choice of the time interval between samplings [51,67]. In the matrix, we also recorded turns because stops are too rare to allow a correct representation of the path followed by the butterfly; indeed, butterflies do not find reasons to stop because the matrix is host plant, food source and mate free. Recording turns is more subjective because it requires a decision of the observer and may lead to oversampling when too many small turns are considered [51]. Serial correlation (autocorrelation) in movement parameters was tested for each path using Pearson's correlation coefficient for move length and circular-circular (T-linear) correlation coefficient (with *P *value estimated by permutation test: [55] pp. 151–153) for turning angle, using lag value from 1 to 5. Few autocorrelations were significant: for move length and turning angle respectively, 1 and 4/55 for FRAG within habitat, 6 and 6/115 for AGGREG within habitat, 2 and 0/40 for FRAG outside habitat, and 9 and 4/90 for AGGREG outside habitat. Furthermore, these few significant autocorrelations appeared at various lags in the various paths (not shown). Consequently, there was no indication that the recording of turns could have biased the data on move length and turning angles.

Finally, the path data analysed in our study were collected in different years (all in June, during the flight season of *P. eunomia*): 1994 for FRAG habitat, 2002 for AGGREG habitat, and 2004 for FRAG and AGGREG matrix. However, as we only focused on adult behaviour, weather conditions are clearly more important than any other kind of year effect. Weather is a major determinant of butterfly activity and behaviour [68], and varies more largely between days of a given years than between years. By restraining path recording to favourable weather conditions, it becomes reliable to compare such data even collected from different years.

## Abbreviations

AGGREG: aggregated patch network (see methods); CRW: Correlated Random Walk; FRAG: fragmented patch network (see methods); GOF: Goodness-of-fit

## Authors' contributions

NS and AJ conceived of the study and collected the data concerning movement outside habitat. NS performed the statistical analyses. NS, HVD and MB wrote the manuscript. All authors read and approved the final manuscript.
